# Higher education students perceptions of NNESTs’ language proficiency: will it affect their learning effectiveness?

**DOI:** 10.3389/fpsyg.2023.1106066

**Published:** 2023-07-13

**Authors:** Xiaoyi Bing, Xiaoqing Gao, Zhen Yang

**Affiliations:** School of Educational Science, Hunan Normal University, Changsha, China

**Keywords:** NNESTs’ language proficiency, students’ perspective, higher education, learning effectiveness, ELT

## Abstract

Increasingly, higher education institutions are giving more attention to the language proficiency of non-native English-speaking teachers (NNESTs) due to their growing numbers. Despite a recent surge in the literature on NNESTs in the global discourse of English language teaching (ELT), the impacts of NNESTs’ language competency within the higher education systems of their countries remain woefully under-examined. Of particular concern is the absence of students’ voices. Therefore, this study explores higher education students’ perception of NNESTs’ language proficiency. Data was collected through class observations of five NNESTs and followed-up semi-structured interviews with five student focus groups recruited randomly from each class. Our results show that while students concur that NNESTs’ language proficiency contributes to their learning performance in class, other factors (e.g., the teacher’s effective teaching style and charming personality, relaxed class atmosphere, the difficulty level of the teaching materials, and the learners’ proficiency level) also perceived to play key roles in boosting students’ class learning effectiveness. The findings highlight the need to include students in the design of teaching approaches, course design, and curricula, as well as the reflection process about NNESTs’ language proficiency.

## 1. Introduction

The considerable and growing numbers of non-native English-speaking teachers (NNESTs) worldwide have been putting increasingly more pressure on English language teachers and how they are trained (Freeman et al., 2015). In particular, concerns regarding the quality, ability, and language proficiency (LP) of trained teachers or educational professionals are especially widespread in non-English-speaking nations, where communicative competence in English is regarded as crucial for success in the global economy (Bulter, 2004; Graddol, 2006). According to Bolton (2004), there are over 500,000 secondary school English teachers in China alone, owing to the early inclusion of teaching foreign languages in school curricula (Elder and Kim, 2014). The ever-increasing number of NNESTs has prompted an evaluation of how NNESTs’ English proficiency is measured and perceived (Eslami and Harper, 2016). However, despite a recent increase in studies on NNESTs in the global discourse of English language teaching (ELT) (Hayes, 2009), researches to date have largely focused on the definition of teachers’ LP, the impact of teachers’ LP on their teaching practice and self-efficacy as well as teachers’ measured and self-measured LP and its impact. Limited researches have looked into students’ perceptions of NNESTs’ language proficiency. Thus, our study sought to explore students’ perceptions of NNESTs’ language proficiency and how students perceive the effect of NNESTs’ language proficiency on their learning effectiveness in class. It differs from the previous studies in that teachers’ language measurement and its effect in this study is mainly conducted from the perspective of students’ views. This study is a qualitative research encompassing class observations and post-observation interviews, whose aim is to determine the extent to which NNESTs’ language proficiency contributes to students’ class learning effectiveness from students’ points of view. The result from this study can be used to generate an understanding of students’ experiences under NNESTs’ instruction and provide valuable insights for the development of NNESTs’ teaching effectiveness.

## 2. Literature review

### 2.1. The construct of language proficiency (LP)

Current theories of proficiency tend to include components of language competence or the context of language use. From one perspective, teachers’ LP encompasses not only general LP in the context of both formal and informal communication but also specialist communication skills including good command of specialized terminology and high discourse competence (Elder, 2001; Elder and Kim, 2014). From another perspective, Freeman et al. (2015) introduced the notion of English-for-Teaching through re-conceptualizing teachers’ LP. In their theory, teachers’ LP is not perceived as one’s general English proficiency but as a specialized subset of language skills required to prepare and teach lessons, for instance, managing the classroom, understanding and communicating lesson content, as well as assessing students, and providing them with feedback. Besides, Richards et al. (2013) included teachers’ LP in teachers’ subject knowledge, thus highlighting LP as an essential component of effective teaching. Finally, Andrews first introduced (1999, 2001, 2003) the concept of teacher language awareness (TLA) and correlated it with pedagogical content knowledge (PCK) coined by Shulman (1987). Andrews demonstrated in his research (2001, 2003) that TLA bridges teachers’ pedagogical content knowledge with their communicative language ability. Specifically, one language teacher’s LP involves not only his/her knowledge about language but also knowledge of language.

Universally, various language proficiency tests of different types have been developed to measure teacher language proficiency in various contexts (Baocheng, 2006). The earliest language proficiency scale was the *Foreign Service Institute Scale (FSI)* developed by the US government in 1955, which pioneered the development of the language proficiency scale (Alderson, 1991; North and Schneider, 1998). Setting out from it, multiple language proficiency scales have appeared in Europe, the United States, Canada, Australia, and other regions, such as the *American Council on the Teaching of Foreign Languages Oral Proficiency Interview*, (*ACTFL OPI*, American Council on the Teaching of Foreign Languages, 2002) a commonly utilized oral proficiency test tied to the ACTFL guidelines (Elder, 2001), *International Second Language Proficiency Ratings* (ISLPR, Ingram and Wylie, 1991; Wylie and Ingram, 1999) including the specified purpose model and the general proficiency model, targeting at different pragmatic situations, the *Canadian Language Benchmarks (CLB, Pawlikowska-Smith, 2000)* consisted of speaking and listening module, reading module as well as writing module and the most influential one, the *CEFR (Common European Framework of Reference for Languages: Learning, teaching, assessment)* jointly developed by more than 40 member states of the Council of Europe (2001).

As described above, the term “proficiency” is defined differently in various settings, and the standards for proficiency vary (Dogancay-Aktuna and Hardman, 2012). Thus, the difficulties of defining and measuring language proficiency in general and consequently teachers’ LP (Van Canh and Renandya, 2017) bring challenges when performing this type of research.

### 2.2. The impact of teachers’ language proficiency

As Bolton pointed out in 2004, teachers’ LP not only influences teachers’ confidence, teaching skills, and content, but students’ motivation and learning effectiveness. With the increasing numbers of non-native-speaker teachers (NNST) employed, especially in non-English-speaking countries (Elder and Kim, 2013), the impact of teachers’ LP on classroom practice has drawn great attention. However, current researchers mainly focused on the impact of teachers’ LP on their teaching practice as well as self-efficacy, and few studies examine its influence on students’ learning.

Theoretically, a high level of LP has been recognized widely as an important qualification for successful English teaching (Bulter, 2004) and teachers with a high level of LP are believed to be using the target language more confidently and accurately in class (Schulz, 1999). However, Freeman et al. (2015) expressed concerns about some English teachers’ substandard LP due to the ever-increasing number of teachers of English. To investigate the impact of teachers’ LP on their teaching practice, Richards et al. (2013) observed seven non-native language teachers who teach French (*n* = 3), Spanish (*n* = 2), German (*n* = 1), and Japanese (*n* = 1) in a New Zealand high school and found out that teachers with low levels of LP failed to consistently provide meaningful explanations of vocabulary or grammar to students in the observed lessons. In another study conducted by Medgyes (2017), vocabulary, speaking, and oral fluency were considered to be the most problematic areas of NNESTs. These deficits may affect teachers’ ability to correct learners’ errors, manage classrooms, simplify their language according to learners’ levels, and choose proper instructional materials (Medgyes, 2017; Richards, 2017). Moreover, NNESTs with low LP may have the tendency to resort to their first language (L1) out of anxiety that they will not be able to handle problematic situations due to the lack of authentic communication abilities. Tsang (2017) examined the relationship between native English teachers (NETs) and Non-Native English Teachers (NNETs)’ general LP and their teaching effectiveness operationalized by learners’ engagement in Hong Kong’s secondary schools. The results uncover that a high command of English is crucial to both NETs and NNETs, but after a certain proficiency level is reached, higher language standards become less significant in comparison to other aspects that affect teachers’ teaching effectiveness. Similarly, Sadeghi et al. (2019) explored the impact of NNESTs’ LP on how an NNEST teaches a lesson in private institutes in Iran. Once again, they confirm the importance of non-native speakers’ (NNS) language proficiency in their personal teaching performance.

These discussions also extend to how teachers perceive their own language proficiency, which has significant effects on their sense of self-efficacy (Faez et al., 2021). In addition, most researches are conducted among NNEESTs, who often have been noted with lower confidence in their oral language capabilities and language fluency (Árva and Medgyes, 2000). To illustrate, Chacón (2005) examined NNESTs’ self-efficacy beliefs in Venezuela and suggested that the more proficient they perceive themselves to be in speaking, writing, listening, and reading, the higher their self-efficacy will be. Similarly, Eslami and Fatahi (2008) found that teachers’ self-efficacy is positively correlated with their self-reported English proficiency. Choi and Lee (2016) concluded that teachers’ language proficiency and their self-efficacy are interdependent and magnify each other’s impact on teachers’ teaching practice. Faez and Karas (2017) also proved that teachers’ self-efficacy beliefs regarding their pedagogical abilities are correlated with their self-reported language proficiency.

When it comes to the impact of teachers’ LP on students’ learning, Carroll (1975) believes that students’ language learning was significantly influenced by teachers’ language proficiency and the amount of target language (TL) used by the teachers in class. Turnbull’s (2001) study has yielded similar results: maximized and optimal target language (TL) use allows teachers to draw a positive effect on learners’ language learning. However, although language has been brought up frequently in the NNEST literature, limited researches have explored the impact of NNESTs’ language proficiency on students’ learning effectiveness in detail. One possible reason lies in the difficulties of measuring students’ overall learning effectiveness, which requires long-term follow-up of class performance or academic performance.

### 2.3. Students perceptions on teachers’ language proficiency

Most English language teachers’ level of language proficiency is often a matter of concern for them and their employers who link higher levels of language proficiency with higher teaching effectiveness (Faez et al., 2019). However, to aid educators and teachers in achieving classroom success, it is of great importance to investigate students’ perception of their teachers’ LP because students are the ones who spend the most time in classrooms observing teachers (Camburn, 2012). Therefore, students’ perceptions can provide insights into the influence teachers have on students’ development of knowledge, motivation, and engagement in language classrooms (Min and Chon, 2021). Tsang’s (2017) interviews showed students’ coherence with the necessities of NESTs and NNESTs to reach a certain level of language proficiency. On the one hand, teachers with a high standard of English proficiency receive students’ respect and compliments. On the other hand, native-like teachers bring difficulties for weaker learners to understand teachers’ articulation and instructions in class. However, previous studies conducted in the context of students’ perceptions focus mainly on how students perceive teachers’ teaching skills, classroom performance, and feedback (Jamil et al., 2010; Freeman et al., 2015; Mohammad and Rahman, 2016; Min and Chon, 2021) in improving teaching practice, yet failed to emphasize students’ views on the impact of teachers’ drawn on classrooms.

As described above, the limited research on NNESTs’ language proficiency has largely focused on the definition of teachers’ LP, the impact of teachers’ LP on their teaching practice and self-efficacy as well as teachers’ measured and self-measured LP and its impacts on their teaching effectiveness. Little is known about the relationship between teachers’ LP and learners’ learning effectiveness. Building on the limited previous research, the present study is attempt to examine student perception of NNESTs’ language proficiency and how students perceive the effect of NNESTs’ language proficiency on their learning effectiveness in class. Considering the significance of students in the language learning classes, our study attempts to answer the following questions: *(1) What are the students’ perceptions of more/less proficient NNESTs? (2) To what extent do students perceive the influence that more/less proficient NNESTs draw on their language learning effectiveness?*

## 3. Materials and methods

### 3.1. Context

To better understand students’ perceptions of their NNESTs’ LP, we recruited five NNESTs and five focus groups comprising first-year undergraduate students at two comprehensive universities in China. Among the participants, there were two teachers from a Project 211^1^ University, where the author is currently located, and three teachers from a non-governmental university, where the author used to work at (see Table 1).

**Table 1:** Characteristics of the five NNESTs.

Teacher code	Gender	Age	Teaching	Qualification
A	F	43	20	MA in ELT
B	F	31	5	MA in Tr
C	M	28	1.5	MA in Tr
R	F	39	9	MA in Eng Lin
E	F	38	14	MA in Eng Lit

The whole research procedure can be divided into two sections: observation and post-observation group interview. For observation, the researcher (observer) attended each class to estimate the NNESTs’ language proficiency and evaluate students’ class engagement. For post-observation group interviews, 10 students who were of different genders were recruited randomly from each class (50 participants in total) and received class participation points (see Table 2).

**Table 2:** Characteristics of students in the five focus groups.

Group code	No./Gender of students	Students major
A	4/M; 6/F	Education
B	5 M; 5F	International Trade
C	6 M; 4F	Information Engineering
R	2 M; 8F	Music
E	3 M; 7F	Dance

Although teachers and students under our observations and investigations came from different colleges and majors, all classes were conducted based on the same textbook, New Horizon College English 1, which is the most used college English textbook that focuses on English reading and writing skills drilling for first-year students. To observe a wider range of teachers’ use of language for different purposes, observations were purposely made in reading classes only, which normally include at least two lectures with roughly the following class activities. To clarify, the reason why we involve reading classes rather than writing ones is that the former including more question elicitation, knowledge delivering, and interactive discussion involves more linguistic performances of NNESTs and more engagement of students, which are ultimately conducive to the effectiveness of our class observations and teachers’ LP rating.

*Lecture One:*

1. Warm-up (topic-related oral questions raising)
2. Lead-in (background information elicitation)
3. Global reading and detailed reading (text structure analysis and main idea catching)

*Lecture Two:*

1. Language focus (significant phrases and sentence study and appreciation)
2. Critical thinking (topic-related questions provoking)
3. Summary and assignment

### 3.2. Data collection

#### 3.2.1. Measurements of NNESTs’ general LP

**Figure 1:**
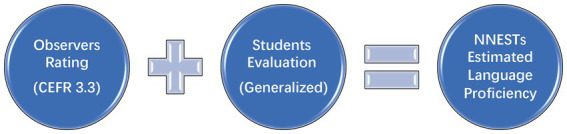
Teachers’ estimated language proficiency.

To investigate whether NNESTs’ LP affects students’ learning effectiveness, defining teachers’ LP is imperative. However, operationalizing teachers’ exact LP is difficult (Richards et al., 2013) as there is no valid and universal LP test available so far (Tsang, 2017). Thus, it was more practical to estimate teachers’ general LP. Notably, many previous studies on teachers’ LP employed the self-rating method for measuring teachers’ LP. However, As Trofimovich and his fellow researchers (2016) claimed that self-assessments of proficiency can be inaccurate and should never be utilized for high-stakes placements. This method suffers from obvious shortcomings in that teachers are inclined to either underestimate or overestimate their exact LP in real cases. Also, considering the main purpose of the current study is to better understand college students’ perception of their NNESTs’ LP, we decided to exclude teachers’ self-estimation of their LP and hand the job to the other two parties: learners and the class observer. Thus, the instruments applied in the study were class observations and students’ evaluations (see Figure 1).

For class observations, we observed 10 college-level English reading classes of five NNESTs, two by each one. Each lesson lasted for 45 min and was audio-recorded for analysis. Before each class, the teacher’s permission was secured in the first place while students were not informed beforehand of the observation to ensure capturing the real class performance of students. Teachers were also reminded to give lectures fully in English while Chinese could be acceptable occasionally when they felt necessary to guarantee sufficient study samples. The researcher was present in the class to evaluate teachers’ overall language performance. As for the indicators of teachers’ LP, we focused on their spoken performance in class because spoken language plays the most vital role in a teacher’s language competence (Sešek, 2007) and most learning in English classrooms is accomplished through teachers’ spoken production. Also, to minimize the impact of inaccurate judgments from the research observer, we introduced the *Common European Framework of Reference—Qualitative aspects of spoken language use (CEFR 3.3)* as a tool to assist the observer in assessing teachers’ qualitative aspects of spoken language use. To clarify, there are two reasons why we choose the CEFR 3.3 as the main tool to evaluate our NNEST’s language proficiency: First, as mentioned in the literature review, the CEFR is now universally recognized as the most influential tool in describing language proficiency levels; Second, CEFR 3.3 focuses on different qualitative aspects of language use and was designed to assess testees’ spoken performances, which agrees with our measuring purpose. Following the indicators in CEFR 3.3, the observer rate teachers from five aspects according to each level descriptor.

In terms of students’ evaluation, 50 students from five focus groups were interviewed and were first asked to look back upon their teachers’ English grammar, pronunciation, expressions, and fluency and then summarize their teacher’s LP using adjectives like “very good,” “not bad,” “not well” and etc. Sophisticated as college students tend to be, possibly, some of them did not want to speak ill of their teachers, but their attitudes could be inferred in the follow-up interviews. Besides, their evaluations were later found to be nearly in line with the class observation results. Therefore, our students’ evaluations of teachers’ LP indeed have a certain reference value.

Through class observations and students’ interviews, we draw a rough depiction of the five NNESTs’ language proficiency. For the convenience of later discussion, we finally categorized them into five classes: high, high-medium, low-medium, and low relatively (see Table 3).

**Table 3:** NNESTs’ measured language proficiency.

Teacher code	Rater-rated spoken range	Rater-rated spoken accuracy	Rater-rated spoken fluency	Rater-rated spoken interaction	Rater-rated spoken coherence	Students’ evaluations (generalized)	NNESTs’ estimated LP
A	C2	C1	C2	C1	C2	Native	High
B	C1	C1	C2	C1	C1	Very good	High
C	B2	C1	C1	B2	C1	Quite good	High-medium
D	B1	B1	C1	B2	B2	Good	Low-medium
E	B1	B1	B2	B1	B2	Good	Low

#### 3.2.2. Post-observation interview

To obtain relatively impartial views on the teacher and the lessons, ten students were called together randomly to take a group interview after the last observed lecture. Each interview lasted about 20 min. The interviews were semi-structured and conducted in Chinese, then audio-recorded and transcribed into English. Besides, key points were specifically noted down during the interviews in case of any overlook. Before discussing their teachers’ LP, teaching practice, and class effectiveness, students were first asked to answer questions such as: Is it important for college NNESTs to possess a high command of English? Will more/less proficient NNESTs affect your learning during the class? And what makes you motivated/demotivated in English classes? Altogether, the collection of data yielded over 30 pages of text, which was later analyzed synthetically using content analysis.

## 4. Results

Most previous studies on teachers’ LP have paid great attention to how teachers perceive their language competence and its impact on their teaching effectiveness. In our study, we attempt to unveil how students perceive their teachers’ LP and its impact on their learning effectiveness. To fulfill the task, we measured five NNESTs’ estimated language proficiency and compared the result with students’ interview data and class observation details. The synthesized results are as follows.

### 4.1. NNESTs’ LP

The participant’s estimated LP levels are shown in Table 3 based on the findings from observation and interview.

### 4.2. Class observations and interviews

#### 4.2.1. TA’s class

TA was one of the two oldest teachers we observed, who had over 20 years of language teaching experience. She ranked first mainly because the words and phrases she resorted to during the class were almost native-like, which might be related to her many visits to overseas top universities. Also, she constructed coherent sentences with very few grammatical errors captured. Further, TA did very well in shifting her instructional tone and explaining grammatical rules in English.

After observing the two separate classes taught by TA, we found that students behaved less motivated and interested in the second class. The reason behind this trend might lie in the variation in teaching content and teaching methods. During the former class, TA chose to raise topic-related oral questions consecutively and randomly selected students to stand up and share their opinions. Thus, the tense atmosphere propelled most students to pay close attention to the teacher’s words. Nevertheless, in the second class observed, key language points in the text were taught through TA’s monologue and only a few students followed her lead with most students being quiet. TA’s students were from a Project 211 University, which means they defeated tens of thousands of students in the Chinese college entrance examination. This can partially explain why TA did not explain or repeat her native-like speech as she took for granted that her students could catch whatever she was talking about. However, she seemed to neglect the fact that most of her students had limited access to native-like colloquial English input before college because of the test-oriented nature of Chinese elementary education.

During the post-observation interviews with students from the first focus group, all of them agreed with the point that NNESTs should have a high level of LP. Interestingly, however, two interviewees mentioned later that TA’s native-like language posed a challenge for them to comprehend the teaching content: *“Well, I cannot fully understand her utterance. She spoke in English all the time. Apart from me, none of my desk-mates understood her, so we gave up.”* and *“Sometimes, I do not understand what she is saying and I feel really frustrated. I think maybe she can speak more Chinese, especially when she asks questions.”* When asked why some of them failed to focus on the teacher’s lecture in the second class, interviewees replied: *“Because I did not even catch up with the first class, so I think there is no need for me to listen to the second one.”* Another student reported: *“To tell the truth, I do not really want to listen to any of the vocabulary explaining or grammar, I hope our class can focus more on the training of our practical English ability, like listening and speaking.”* One interviewee agreed instantly and explained that he hopes he can acquire some ‘practical knowledge’ in the English class; for example, he thinks TA can share some of her traveling experience in foreign countries or introduce some cultural differences between China and English-speaking countries.

From the interviews and observations, we found that the first focus group’s attitudes toward teachers’ LP are contradictory and intertwined. On the one hand, they believe that NNESTs are supposed to master the language they teach and they admire the native-like spoken English of their teacher. On the other hand, they reckon that teachers with high LP should pay more attention to accommodating their students’ language capability when they organize their words because teachers’ ceaseless native utterances may not only jeopardize their learning enthusiasm but decrease their learning effectiveness during class. Also, we found that the inquiry-based teaching method can boost students’ class engagement, while the teacher who uses teacher-centered instruction can fail to grasp students’ attention no matter how “native” his/her language is. According to the observer, the selection of teaching content can also influence students’ class performance. The second reason why TA failed in arousing students’ learning interests in Lesson Two was that she over-emphasized the vocabulary and syntax learning that her students are bored with.

#### 4.2.2. TB’s class

TB was the most energetic teacher among all teachers observed. With respect to her LP, she did quite well in all five assessment criteria, except for one consistently used sentence pattern: *“I would like to…,”* which was repeated too many times and attracted the observer’s attention. During the class, she provided translation thoughtfully after each English sentence she uttered, which is highly accommodating to students with low English proficiency.

During the first class, the observer noticed that, instead of putting much emphasis on the passage structure analysis as well as the main idea learning like TA, TB involved only key learning points from the passage and enriched the text explanation with inspiring critical thinking questions. Meanwhile, in the second class, instead of planting all essential words and phrases into the students’ minds, she offered several hands-on word and phrase practices. In class, students were motivated because of the flow of questions and quizzes. While the teacher acted more like a “host.” Her individual teaching style might be correlated with her rich IELTS teaching experience, the international English test in which she scored band 8.

The interviewees praised TB for the relaxed and pleasant atmosphere she created in every class: *“I enjoy TB’s classes because I do not feel much pressure. Unlike the English class we had back in high school, we are now given time in the class to reflect on the knowledge ourselves. Although the learning pace seems to be slowed down, we feel we have learned more.”* When it comes to discussing the influence of a teacher’s LP on the class, eight interviewees believed it is important for a teacher to have a high LP, while one interviewee disagreed and the other chose to be neutral. One interviewee stated: *“I believe it is important for teachers to have high language proficiency because the higher the teacher’s language proficiency, the more stimulated I will be. Thus, I will be affected. On one occasion, there was a substitute teacher here and I could not understand a single word she said. It seems like she was using a completely different language. We missed TB very much at that time.”*

Considering the result of observations and interviews comprehensively, we found that although TB’s language proficiency is not as native as TA’s, TB’s student-centered class received students’ broader participation. To interpret, instead of acting on her own like TA, TB invited students to play lead in class discussions and practices, which give few opportunities for students to be absent-minded. Second, TB interpreted each English sentence she made, which indicates that she takes account of all students with varied language capabilities. Thus, TB’s interactive teaching strategy and translation support contribute to her students’ favor. Besides, we can conclude that TB’s student-oriented pedagogical strategies and translation support in engaging most students appear to be the fundamental keys to the success of her classes.

#### 4.2.3. TC’s class

TC was the youngest of all teachers and had one and a half years of teaching experience by the time of the study. Equipped with two certificates of the China Accreditation Test for Translators and Interpreters of level two (CATTI)*, TC’s overall language performance was fairly good.

Considering the needs of students with low English levels, TC arranged a series of group work activities in each lesson and invited at least one group to demonstrate what they came up with, to add more fun to his English classes. According to the observations, when activities were held, most students would raise their heads and follow the lead. However, when it came to passage reading or language points learning, only very few students would still follow the teacher and be responsive.

When the observer asked interviewees why some of them behaved so differently in different learning stages, one active interviewee explained that: *“The passage in the textbook we learn right now is too difficult for me. I always feel like there is a huge gap between the knowledge I possess and the knowledge teacher imparts, so I have to preview the text at length before each class. However, sometimes I forget to preview the content, so I choose to be quiet.”* Some of the other interviewees agreed with these remarks and communicated that the textbook, *New Horizon College English 1,* is too hard for them: *“We were STEM students back in high school and English is not our strong suit.”* However, when the interviewees are asked to evaluate TC’s language proficiency, they spoke highly of TC’s pronunciation and expressions. Also, some mentioned that TC’s gentle while humorous personality and fashion sense always draw their attention in class: *“TC is good at telling jokes, and the clothes he wears every day are clean, tidy, and fashionable.”*

Based on the aforementioned observations and remarks, we noted that apart from teachers’ English proficiency, their appealing personality and physical attractiveness can also contribute to students’ engagement in class. Besides, as revealed in the observation, the difficulties of teaching materials can draw an unexpectedly huge impact on students’ willingness to learn during the class, especially for students who majored in science and engineering.

#### 4.2.4. TD’s class

TD was an experienced teacher with a total of 9 years of English teaching experience and 3 years of art-students teaching experience. Working with art students for several semesters, TD is quite familiarized with art students’ characteristics. TD was rather tough in class and established a rule that anyone who answered her question, whether it was correct or incorrect, would receive points toward his or her final grade. This was done to guarantee that the art students were actively participating in the class.

To ensure art-students’ participation in the class, TD behaved rather tough in class and set up a rule that anyone who answered her question, whether it was correct or incorrect, would receive points toward his or her final grade. As a result, TD’s students behaved much more energetically and passionately than students in any other observed classes. However, in terms of TD’s language proficiency, we detected a few incompetence in grammar and phonology. For instance, she forgot to apply “s/es” in the third person singular several times: “*The author use figurative language to…,*” “*The government tell us to…*” Also, influenced by her local accent, TD could not distinguish between the pronunciation of/n/and/I/; for example, the word “life [laɪf]” is pronounced more like “knife [naɪf]” and “globalization [ˌgləʊbəlaɪˈzeɪʃn]” is pronounced more like “[ˌgləʊbənaɪˈzeɪʃn],” which hindered the observer’s understanding to her expressions sometimes.

However, when interviewees were invited to evaluate TD’s LP, comments as “It’s fine” and “pretty good” were made frequently. It seemed that students were unmindful of TD’s grammatical and pronunciation mistakes. Instead, they appreciated TD’s classes. For example, one commented that: “We like TD’s English class because, in her class, I can accumulate a large number of useful words and sentences. For example, I have been learning CET-4* myself recently, and surprisingly I found a lot of words that I learned the other day in TD’s class.” “This is true,” according to the observer. In TD’s class, CET-4 was frequently brought up and the class seemed to be quite test-oriented. For example, when TD went over the core vocabulary of CET, she would highlight the words and ask students to memorize them right away. This pragmatic teaching style was widely welcomed by TD’s students. This is perhaps due to students’ familiarization with the test-driven learning environment from their high school years.

From the above interviews and the observations, two inferences can be made as to why TD’s students have no problem with her grammatical and phonetic flaws. First, the reward system she established in class captures the attention of art students. Fear of failing the fail exam, most students, if not all, are willing to pay attention to any opportunity to score in the class. Second, under the influence of the “Certificate Craze” in China, students may find TD’s examination-oriented classes extremely beneficial, which motivates them to be more attentive in class.

#### 4.2.5. TE’s class

TE was the second oldest teacher we observed and have been teaching English for over 14 years at the time of our research. Interestingly, TE used to work in a vocational college and had just joined the school 3 months ago.

In her class, frequent oral mistakes were detected. For example, sentences without articles occurred frequently, such as “He does not have job currently,” “English as universal language,” “Mathematics is language of …” What is more, she also failed in correcting students’ mistakes in article omission. Moreover, the observer noted her omission of “s/es” when using third-person singular forms of verbs and other mistakes in verb tenses. Also, it appears that TE’s First Language (L1) has an impact on how she pronounces words, which sometimes made it hard for listeners to understand. Apart from grammatical mistakes and pronunciation incompetence, our observer noticed that TE occasionally had difficulties in converting what she wanted to express into the target language (TL). Instead, there would be silence all of a sudden or she would use Chinese immediately. For instance, TE produced this sentence in the discussion about why people nowadays are insatiable and want to buy everything they can get their hands on: “*The reason why people want to buy everything they can get is that there are advertisements on the … on the …* (Chinese applied instantly).” Two-thirds of the students in TE’s class were quiet and it seemed like TE was already accustomed to the quietness and proceeded in her class anyway.

However, the post-interview results were polarized. When asked about the reason why some of them acted very quietly in class, one interviewee explained that: *“It seems that each class follows the same pattern, so it is a little bit boring. Besides, I am not good at English. So, you know.”* As for interviewees who followed the class closely, they claimed that they like TE because she is an experienced teacher, and they like TE because of her charming personality: *“TE cares about us very much. Once I caught a cold, she told me not to sit under the air conditioner. Although she is older than us, there is no generation gap between us. We have a lot of common topics (to talk about) together.”* When the interview proceeded to TE’s language proficiency evaluation, the interviewees believed that their teacher’s LP was “good” and “okay.” However, when asked, “Do less proficient NNESTs affect your learning in the class?,” the interviewees agreed on statements like: *“If the teacher’s language proficiency is not high, the class learning atmosphere will be jeopardized.”* and *“(High language proficiency is necessary) because English teachers need to make sure their students understand them. Besides, they are college English teachers, so their English level must be higher than our high school teachers.”*

Putting the observation results and interview outcomes together, when we look at TE’s overall teaching success it turns out that the least proficient teacher E (whose LP were rated lower than teacher A, B, and C) was an effective teacher in her students’ perspective. For instance, without mentioning her grammatical errors or her toneless voice, TE’s students talked highly of her extensive teaching background and kind nature. The outcome suggests that while teachers’ LP may help with their effective instruction, there are undoubtedly other factors at play that are unrelated to language competency. To interpret further, students care about the teacher’s language proficiency, but once it reaches a certain level, other factors can also have a huge impact on students’ engagement.

## 5. Discussion and implications

The aim of this small-scale case study was to fill the gap in the extant literature regarding NNESTs’ language proficiency by exploring students’ perceptions of their NNESTs’ language proficiency and how students perceive the effect of NNESTs’ language proficiency on their learning effectiveness in class. To investigate, we observed five English teachers who teach the same language course in different classes. Based on the data analyzed, students agreed with the significance of NNESTs to have a high language proficiency. Besides, other non-language proficiency factors that influence their learning effectiveness in class are also brought up by students.

The results of the study firstly show students’ agreement with the statement that high language proficiency is important in delivering an effective language lesson. According to students, teachers with high LP can be role models in stimulating students’ English learning enthusiasm. On the contrary, teachers with limited LP can make students disappointed with themselves for not understanding most teachers’ instructions. Besides, we found that interviewees tend to harbor a higher “expectation” for college English professors in terms of their language proficiency, which is thought to be superior to that of other teachers from primary and middle school teachers. These findings offer support for the proposition that teacher language proficiency contributes to the effective delivery of the lesson (Andrews, 2003; Richards et al., 2013; Medgyes, 2017; Faez et al., 2019). Secondly, based on the observation results and feedback from students, it is interesting to notice that teachers with a high level of LP did not deliver the most effective class, while teachers who were categorized with lower LP did not completely fail in capturing students’ attention. According to students with poor language proficiency, teachers’ non-stop local output can sometimes hamper their interactions with teachers, thereby decreasing their class learning effectiveness. This statement indicates that although students attach great importance to teachers’ LP, they do not perceive teachers’ high level of LP as the only decisive factor in the success of language class. Whereas, students revealed that other non-LP factors also have an impact on how well they learn in NNESTs’ classes. To illustrate, they believed that the teacher’s effective teaching style, charming personality, and even appealing appearance, relaxed while interesting class atmosphere, the difficulty level of the teaching materials, as well as their own English proficiency level all contribute to how involved they are in class. From students’ perspective, English teachers, no matter proficient or not, should accommodate all students’ language proficiency levels. Otherwise, less proficient students may quickly lose interest in the class. Also, students believe that the difficulties of teaching materials have an impact on how engaged they are in learning throughout the class. In fact, they yearn for learning materials that not only suit their learning capabilities but also for practical knowledge they resort to in tests or daily communication. What is more, during our observations, students are found to be more motivated in classes where effective teaching strategies are implemented. For instance, most students’ attentions are observed to be greatly aroused when teachers raise open-end questions from time to time. It has also been noticed that students enjoy the relaxed class atmosphere created by humorous, energetic, or easy-to-approach teachers. In this case, students seem to be unmindful of less proficient teachers’ inauthentic pronunciation or expression. In other words, students do not perceive NNESTs’ LP as the only benchmark in defining a successful class but mind other factors that facilitate their learning outcomes.

The findings of this research are not unexpected given the feedback to NNESTs and their faculties and it does draw teachers’ attention to include students’ voices in their professional development, design of teaching approaches, course design, and curricula, which are frequently overlooked within the higher education sector (Bovill et al., 2011). Compared with junior high or high school students, Chinese college students seem to behave more slacked off because they no longer face the huge pressure of Gao Kao. Thus, to stimulate students’ class learning effectiveness, teachers need to take more external factors into consideration other than prompting their LP only. Firstly, native-like NNESTs should pay more attention to accommodate students’ varying levels of English proficiency in class. One possible solution is to use mixed languages-Chinese and English-to equip students’ understanding. Secondly, due to college students’ thirst for information that goes beyond textbooks, or what they call “practical knowledge,” NNESTs should be more selective when choosing their teaching materials to meet students’ practical demands and leave more space for students to explore both individually and collectively. Besides, some students who participated in the interview voiced complaints and concerns about the New Horizon College English 1 textbook’s usability, which they believe is to blame for their failure to pay close attention during lectures. The comments do lead us to question whether it is practical for Chinese higher education institutions to adopt uniform English teaching materials while dealing with students at various proficiency levels, which we believe is the next issue to be covered. Thirdly, NNESTs can use various teaching techniques with various students to increase the learning efficiency of the class. For instance, Rahman and Yuzar’s research (2020) strongly advised language teachers to take their students’ preferences into account when choosing teaching approaches. Last but not least, although NNESTs with lower levels of LP can achieve some aspects of effective language teaching in the classroom, they should never stop improving both their linguistic proficiency and pragmatic competence to maximize the learning experience of students (Nemtchinova et al., 2010). Professional development support targeted at NNESTs’ teaching needs and particular to the realities of their everyday classroom teaching should also be provided.

As Eslami (2020) noted, open discussion of NNESTs issues which involves stakeholders from the field in finding ways to address the issues is highly necessary. By including students’ voice and needs into consideration, the results that have been reported help NNESTs understand how students view their LP in the classroom. From students’ perceptive, in addition to improving their oral LP, NNESTs should focus on developing their pedagogical teaching abilities, teaching materials selection, and classroom learning atmosphere.

## 6. Limitations

This study has attempted to explore beyond teachers’ own judgment of their language proficiency and students’ learning effectiveness in class. In contrast, we investigated how students perceive teachers’ LP and its effect on their process of learning, which highlights the need to engage students in the reflection process about English language teaching. However, the limitations of this study cannot be neglected.

First, we observed and interviewed first-year college students only to control the variables of teaching materials. However, controlled research with separate age groups (first and second-year students) would be preferable in ensuring the reliability and generalizability of the results. Second, the participants in our study were only chosen from two institutions, thus it is unknown whether data from other samples in other universities, particularly from a different country with different teaching approaches, will provide the same results. Thus, a wider population needs to be studied in future studies. Lastly, this research was conducted primarily with a limited number of observations. Considering the fact that an observer’s presence in the classroom could have affected the teachers’ performance as well as students’ engagement, future studies should consider involving more observation sessions with a longer period.

## 7. List of non-standard abbreviations

1. CATTI: China Accreditation Test for Translators and Interpreters, which is a state-level vocational qualification examination entrusted by the Ministry of Human Resources and Social Security (MHRSS) of the People’s Republic of China and implemented and administrated by China International Publishing Group (CIPG). Included in the list of vocational qualifications of China’s State Council, CATTI is the most authoritative translation and interpretation proficiency accreditation test, which has been implemented throughout the country according to uniform standards and in compliance with the national system of professional qualification certificates (Retrieved from https://www.catticenter.com/cattiksjj/1848).
2. CET: The College English Test is a national test of English as a foreign language in the People’s Republic of China. This test includes two levels: Band 4 (CET-4), and Band 6 (CET-6), which are designed to examine the English proficiency of undergraduate and postgraduate students in China and ensure that they reach the required English levels specified in the National College English Teaching Syllabuses (NCETS) (Zheng and Cheng, 2008).

## Data availability statement

The datasets presented in this article are not readily available because the data can only be accessed by the authors. Requests to access the datasets should be directed to 1505279254@qq.com.

## Ethics statement

The studies involving human participants were reviewed and approved by Hunan Normal University (2022549). The patients/participants provided their written informed consent to participate in this study.

## Author contributions

XB and XG contributed to all stages of the research project and writing. ZY contributed to the design of semi-structured interviews, data interpretation, and writing. All authors contributed to the article and approved the submitted version.

## Funding

This work was supported by Hunan Provincial Education Planning Base Project (grant number XJK23AJD045).

## Conflict of interest

The authors declare that the research was conducted in the absence of any commercial or financial relationships that could be construed as a potential conflict of interest.

## Publisher’s note

All claims expressed in this article are solely those of the authors and do not necessarily represent those of their affiliated organizations, or those of the publisher, the editors and the reviewers. Any product that may be evaluated in this article, or claim that may be made by its manufacturer, is not guaranteed or endorsed by the publisher.

## Footnotes

^1^Project 211 is a project of National Key Universities and colleges initiated in 1995 by the Ministry of Education of the People’s Republic of China.

## Abbreviations

NNESTs: non-native English-speaking teachers
NNSTs: non-native-speaker teachers
NETs: native English teachers
LP: language proficiency
ELT: English language teaching
TLA: teacher language awareness
PCK: pedagogical content knowledge
TL: target language
L1: first language
